# The Landmark Series: Evaluation and Management of Adrenal Incidentalomas

**DOI:** 10.1245/s10434-025-17296-8

**Published:** 2025-04-30

**Authors:** Lily Owei, Heather Wachtel

**Affiliations:** Department of Surgery, Hospital of the University of Pennsylvania, Philadelphia, PA USA

**Keywords:** Adrenal incidentalomas, Catecholamines, Mineralocorticoids, Androgens, Glucocorticoids, Adrenalectomy, Adrenocortical carcinoma (ACC)

## Abstract

Adrenal incidentalomas are adrenal masses ≥ 1 cm discovered on imaging studies for unrelated clinical conditions. The prevalence of adrenal incidentalomas has increased as a byproduct of the widespread use of cross-sectional imaging, particularly in older adults. The clinical significance of adrenal incidentalomas varies based on tumor size, hormonal activity, and imaging characteristics. While most adrenal incidentalomas are benign and asymptomatic, a significant minority are hormonally active or malignant, necessitating careful evaluation and management. Adrenal hormone secretion can have significant clinical implications. Biochemical testing is crucial to assess for hormone excess, including steroid hormones (mineralocorticoids, glucocorticoids, and androgens), which are made in the adrenal cortex, as well as catecholamines, which are made in the adrenal medulla. Non-contrast computed tomography (CT) is the preferred modality for evaluating adrenal nodules as it allows for assessment of tissue density in Hounsfield units (HU). Benign lesions typically have an homogeneous appearance with HU ≤ 10. Contrast-enhanced CT with delayed washout can help differentiate benign tumors from malignant tumors. Tumors ≥ 4 cm, or those with indeterminate features may require further imaging, such as magnetic resonance imaging (MRI) or positron emission tomography (PET)/CT. The management of adrenal incidentalomas is determined by hormonal secretion and imaging characteristics. Surgical resection is recommended for functional tumors and those that are suspicious for malignancy, including tumors ≥ 4 cm in size and those with rapid growth. Non-functional tumors < 4 cm may undergo imaging surveillance. The goal of this review is to summarize the contemporary literature and guidelines on adrenal incidentalomas, and to describe the key principles regarding evaluation and management.

## Epidemiology

Adrenal incidentalomas can be considered both a modern and an iatrogenic diagnosis,  and are defined as adrenal masses ≥ 1 cm discovered on imaging obtained to evaluate unrelated clinical conditions.^1^ The prevalence of incidental adrenal masses varies by data source, patient selection, and diagnostic method, but ranges from 1 to 5% on computed tomography (CT) scans.^2–5^ In autopsy studies, the prevalence of adrenal incidentalomas ranges from 1 to 8.7%, and increases with age.^6,7^ Less than 1% of adrenal tumors are seen in people under 18 years of age, while the prevalence increases to more than 10% in the elderly, with peak increase between 50–60 years of age.^1,8,9^ These prevalence estimates may reflect some selection bias. In a large, unselected population undergoing annual check-ups in China, the overall prevalence was 1.4%, ranging from 0.2% in subjects 18–25 years of age, to 3.2% in those older than 65 years of age.^10^ The prevalence of adrenal incidentalomas does not vary by sex.

With the increased availability and use of diagnostic cross-sectional imaging, there has been a corresponding increase in incidental findings, including adrenal nodules.^7,11,12^ This increase is partially attributable to the more frequent discovery of small adrenal masses in older adults. One retrospective, population-based cohort study in Olmsted County, MN, USA, from 1995 to 2017 found a tenfold increase in the standardized incidence over the study period.^9^ This increase was primarily driven by the incidental discovery of adenomas <4 cm in size in patients ≥40 years of age. These data are of particular relevance given global trends toward population aging, which would suggest that both the occurrence and the identification of adrenal incidentalomas will continue to rise in the coming decades. It is therefore of paramount importance to accurately identify nodules that present a risk to patient health, both to ensure appropriate management and to minimize overtreatment.

Adrenal incidentalomas can be broadly categorized by two distinct parameters: hormone secretion and risk of malignancy. The reported prevalence of subtypes of adrenal tumors varies significantly due to heterogeneity in study populations and definitions of hormone excess between studies. In one review including 44 studies, benign adrenocortical adenomas accounted for 41% of nodules, metastases from extra-adrenal malignancies 19%, adrenocortical carcinomas 10%, myelolipomas 9%, and pheochromocytomas 8%, with other lesions such as adrenal cysts comprising the remainder.^13^ A prospective, multicenter, cross-sectional observational study including 1005 Korean patients with newly diagnosed adrenal incidentalomas found the vast majority were non-functional adrenocortical adenomas (83.3%); the remainder included cortisol-secreting tumors (4.4%), pheochromocytomas (6.0%), and aldosterone-secreting tumors (6.1%).^14^ A more recent review of 14 studies found that adenomas comprised 80–85% of adrenal incidentalomas. Of the adenomas, 40–70% were non-functioning, 20–50% were associated with mild autonomous cortisol secretion (MACS), 1–4% had overt Cushing’s syndrome, 2–5% were aldosterone secreting, and 1–5% were pheochromocytomas.^15^ Despite the large variability between studies, the general trend is that non-functional adenomas are the most frequent diagnosis, followed by cortisol-secreting, aldosterone-secreting, and catecholamine-secreting tumors respectively, while primary adrenocortical carcinoma (ACC) remains rare. Table 1 summarizes the prevalence of adrenal tumor subtypes based on multiple studies.

**Table 1:** Prevalence of adrenal tumor subtypes

Diagnosis	Prevalence	Refs.
Adrenocortical carcinoma (ACC)	0.3–11%	5, 6, 8, 9, 14, 15, 19, 29, 32, 33, 43, 54–56
Aldosterone-secreting tumor	1.6–11.5%	8, 10, 19, 32, 43
Androgen-secreting tumor	< 0.1–1%	9, 54
Mild autonomous cortisol secretion (MACS)	6–50%	8, 9, 14, 15, 19, 32, 43, 47, 54–56
Non-functional adenoma	40–85%	6, 8, 10, 14, 15, 43, 47, 54, 56
Other benign	6.6–9.4%	9, 43, 54
Other malignant	0.5–8.6%	9, 14, 15, 54
Overt Cushing’s syndrome	0.4–4%	9, 15, 43
Pheochromocytoma	1–14%	5, 6, 8, 14, 15, 19, 29, 33, 43, 54–56

## Biochemical Evaluation

Adrenal hormone evaluation is crucial for the management of adrenal incidentalomas, as functional tumors typically require surgical resection. Biochemical evaluation should be performed in conjunction with a careful clinical assessment including history and physical examination. A summary of the current screening guidelines can be found in Table 2.^15–17^ While prior guidelines have recommended routine testing for all adrenal hormones, contemporary guidelines suggest routine testing for autonomous cortisol secretion and catecholamines, and only selective testing for aldosterone and androgens in patients with clinical signs or symptoms.^15,18^ These recommendations are based on the higher likelihood of clinically occult disease in pheochromocytoma and MACS, which makes testing based on clinical findings less reliable. Selective screening does increase the importance of meticulous and expert clinical evaluation.

**Table 2:** Suggested biochemical testing for adrenal incidentalomas (adapted from the European Society of Endocrinology Clinical Practice Guidelines)^15^

Hormone	Screening test	Cut-off values	Confirmatory tests	Who to test	Clinical manifestations
Cortisol	1 mg overnight DST	Serum cortisol ≤50 nmol/L [≤1.8 µg/dL] excludes autonomous cortisol secretion	24-h urine-free cortisol; late-night salivary cortisol	All patients with adrenal incidentaloma	MACS: Often subtle or absent clinical signs
					Cushing’s: Central obesity, facial plethora, proximal muscle weakness, striae, easy bruising
Catecholamines	Plasma-free metanephrines	2–3 × upper limit of normal is diagnostic	Plasma catecholamines; 24-h urine metanephrines and catecholamines	Patients with adrenal lesions with imaging features not typical for a benign adenoma	Hypertension (paroxysmal or sustained), headaches, palpitations, sweating, anxiety
Aldosterone	ARR	ARR >20 (ng/dL)/(ng/mL/h)	Saline suppression test	Patients with concomitant hypertension or unexplained hypokalemia	Hypertension, hypokalemia
Androgens	Androstenedione, testosterone, DHEAS, 17β-estradiol (in postmenopausal women and men)	Varies by laboratory	Not typically required	Patients with imaging or clinical features suspicious for ACC	Women: Hirsutism, acne, voice changes, male-pattern baldness, menstrual irregularities
					Men: often asymptomatic, gynecomastia

DST, dexamethasone suppression test; MACS, mild autonomous cortisol secretion; ARR, aldosterone-to-renin ratio; DHEAS, dehydroepiandrosterone sulfate; ACC, adrenocortical carcinoma

### Cortisol

Evaluation for hypercortisolism is complex, and clinical practices may vary. Widely used tests include the low-dose (1 mg) dexamethasone suppression test (DST), 24-h urine-free cortisol (UFC), late-night salivary cortisol, and early-morning adrenocorticotropic hormone (ACTH) and cortisol. The preferred initial screening test is the 1 mg overnight DST, as this can both assess for autonomous cortisol secretion and discriminate between MACS and Cushing’s syndrome. Serum cortisol ≤50 nmol/L (≤1.8 µg/dL) after dexamethasone administration is the recommended cut-off value, which has a high sensitivity (95–100%) but low specificity (60–80%) for autonomous cortisol secretion.^15,19^ Patients who fail to suppress serum cortisol on dexamethasone suppression testing but do not exhibit the signs and symptoms of clinically apparent Cushing’s are considered to have MACS, previously known as subclinical Cushing’s.^15–17^ For patients with initial biochemical findings suggestive of cortisol secretion, confirmatory testing with one or more independent assays is required for definitive diagnosis.

Current society guidelines recommend use of the low-dose DST or two measurements of either the late-night salivary cortisol test or 24-h UFC.^16^ However, in patients suspected of MACS, UFC has a sensitivity of only 80–98%.^16,20^ In patients with adrenal incidentalomas and MACS, the sensitivity of late-night salivary cortisol is only 22.7%, suggesting that normal levels do not rule out subclinical hypercortisolism.^21^ Thus, UFC and late-night salivary cortisol may not be appropriate screening tests in patients without overt signs or symptoms of hypercortisolism. Of note, dexamethasone absorption and metabolism may be affected by various medications and disease states. For example, oral contraceptive pills increase cortisol-binding globulin, resulting in a 50% false positive rate.^16^ Therefore DST testing must be interpreted within the clinical context.

### Catecholamines and Metanephrines

While pheochromocytomas are less commonly present as adrenal incidentalomas (1.5–14.0%), there is a growing appreciation that these tumors may manifest in a clinically occult fashion, so-called ‘silent pheos’.^19,22^ While the 2016 European Society of Endocrinology (ESE) guidelines recommended testing to exclude pheochromocytoma in all patients with adrenal incidentalomas, the updated 2023 guidelines recommend testing only in patients with adrenal lesions with imaging features not typical of a benign adenoma.^15^ Initial screening consists of measurement of plasma-free metanephrines, which have the highest sensitivity for diagnosis.^23^ Due to high rates of false positive testing, elevations of two to three times the upper limit of normal are typically used to establish a diagnosis of pheochromocytoma. Indeterminate levels may require further evaluation with plasma catecholamines or 24-h urine metanephrines and catecholamines. Dopamine testing is typically reserved for patients with head and neck paragangliomas, while vanillylmandelic acid (VMA) testing is no longer routinely performed for patients with adrenal lesions.

### Aldosterone

The primary clinical manifestation of hyperaldosteronism is hypertension. Up to 50% of patients also present with concomitant hypokalemia due to the effect of aldosterone on the sodium-potassium co-transporter. Biochemical evaluation includes a basic metabolic panel, plasma aldosterone concentration, and plasma renin activity. An aldosterone-to-renin ratio (ARR) of >20 (ng/dL)/(ng/mL/h) is frequently used for diagnosis.^19^

It is important to note that conventional ARR thresholds likely underestimate the prevalence of renin-independent aldosterone production. The commonly used diagnostic threshold of ARR >30 (ng/dL)/(ng/mL/h) has a low sensitivity for diagnosis of biochemically overt primary aldosteronism (by oral sodium suppression test) in patients across all blood pressure levels (22.2–50.0%), suggesting significant underdiagnosis of primary aldosteronism.^24^ While ARR is considered the standard screening test, these data suggest that liberal use of saline suppression testing might have higher sensitivity for case detection. Current guidelines recommend confirmatory testing for indeterminate cases. Adrenal vein sampling is considered standard of care for subtype differentiation.

### Androgens

While androgen-secreting tumors are rare, the clinical presentation is typically dramatic and unambiguous. Signs and symptoms may include hirsutism, acne, voice changes, male pattern baldness, and menstrual irregularities in women. Androgen secretion is most commonly diagnosed in the context of adrenocortical carcinoma. Therefore, Endocrine Society guidelines recommend measurement of sex steroids and steroid precursors, including androstenedione, testosterone, dehydroepiandrosterone sulfate, and 17β-estradiol (in postmenopausal women and men only) in patients with imaging or clinical features suspicious for ACC.^25^

### Urine Steroid Metabolomics

Urinary steroid metabolomics utilize mass spectrometry analysis of steroid metabolite excretion profiles to discriminate between adrenocortical adenoma and ACC. This technique takes advantage of the fact that the majority of ACCs are associated with elevated androgens and/or steroid precursors.^15^ Reported sensitivity is 90% with specificity of 88%.^26,27^ A large prospective, multicenter validation study of more than 2000 patients evaluated the diagnostic accuracy of urine steroid metabolomics compared with conventional imaging strategies for ACC detection.^27^ Urine steroid metabolomics had a higher positive predictive value (34.6%) than tumor size (19.7%) or assessment of imaging characteristics (19.7%). When combined with tumor size >4 cm and unenhanced CT tumor attenuation >20 HU, the positive predictive value increased to 76.4%.^27^ While urine metabolomic testing is a promising tool, it is not yet widely available and is not recommended as part of routine adrenal nodule evaluation.^15^

## Imaging

Non-contrast, or unenhanced, CT is the preferred initial imaging modality for the evaluation of adrenal incidentalomas.^15,18,28,29^ CT allows assessment of tissue density or tissue attenuation and is essential to assess the likelihood of malignancy in adrenal lesions. An adrenal mass is considered benign if it appears homogeneous and lipid-rich, with a density of ≤10 HU on an unenhanced CT scan.^30^ While standard contrast-enhanced CT is not suitable for distinguishing benign from malignant adrenal tumors, CT with delayed washout is more helpful. An adrenal protocol CT includes an unenhanced sequence followed by intravenous contrast administration with repeat imaging at 60–75 s (venous phase) and again at 15 min (delayed phase).^18^ Injected intravenous contrast washes out of benign lesions more rapidly than malignant lesions.^29^ Specifically, benign adenomas demonstrate an absolute percentage washout >60% at 15 min delay, which is therefore used as a key diagnostic cut-off.

The 2023 ESE/European Network for the Study of Adrenal Tumours (ESNAT) guidelines recommend that the initial step is differentiating benign from malignant adrenal incidentalomas using a non-contrast CT. If non-contrast CT is consistent with a benign adrenal incidentaloma, i.e. homogenous in appearance and HU ≤10, no further imaging is required.^15^ This marks a significant shift from prior recommendations.

Tumor size is a key characteristic that determines management, due to the correlation between tumor size and risk of malignancy. In 2002, the National Institutes of Health (NIH) published a consensus recommendation on adrenal incidentalomas, which classified adrenal tumors measuring <4 cm as having a low risk of malignancy, those between 4 and 6 cm as indeterminate, and tumors ≥6 cm as having a high risk of malignancy.^31^ Multiple international society guidelines continue to use a cut-off size of 4 cm to determine management.^8,15,18,25,32,33^ For indeterminate nodules 1–4 cm in size on CT, either immediate additional imaging (adrenal protocol CT or magnetic resonance imaging [MRI]) or interval imaging in 6–12 months is recommended due to a slightly increased risk of malignancy.^18,32^ Nodules ≥4 cm with indeterminate imaging characteristics are typically recommended for surgical resection.

MRI can provide additional data in the assessment of indeterminate nodules. Most adrenal adenomas (70%) have high cytoplasmic fat and are designated as lipid rich. The chemical shift imaging (CSI) technique can be used to detect intracytoplasmic fat and thus distinguish benign lipid rich adenomas from malignant tumors, as evidenced by a drop in signal on out-of-phase imaging.^29^ The diagnosis of an adrenal adenoma can be made with MRI, with CSI with 81–100% sensitivity and >90% specificity.^34^ Some studies suggest that 62–100% of adenomas with attenuation >10 HU on non-contrast CT can be further characterized as lipid rich using the CSI technique;^35^ however, it is less definitive in lipid-poor adenomas, where CT with washout is more revealing.^36^ Of note, CSI should be interpreted with caution in patients with primary hepatocellular or renal cell carcinoma where both the primary neoplasm and adrenal nodule may demonstrate a drop-out in signal.^36^

F18 fluorodeoxyglucose (^18^F-FDG) positron emission tomography (PET)/CT is rarely used as a primary imaging modality in the evaluation of adrenal incidentalomas. In patients with known extra-adrenal malignancies,^18^ F-FDG PET/CT accurately discriminates between metastatic disease and benign lesions.^18,37^ F-FDG PET/CT may also help identify occult primary tumors in the rare cases where an adrenal metastasis is the initial presentation of a previously undiagnosed malignancy.^15^ Imaging with^18^F-FDG PET/CT may also play a role in staging for patients with suspected ACC or pheochromocytoma; however, radionuclide imaging such as ^68^Gallium DOTATATE PET/CT demonstrates higher sensitivity for metastatic lesion detection in patients with pheochromocytoma.^38–40^

## Biopsy

Image-guided biopsy is not routinely indicated for adrenal masses due to both biopsy-associated risk and poor diagnostic performance. Risks of biopsy include procedural, hormonal, and oncologic. Procedural complications, including hematoma, pancreatitis, and pneumothorax, occur in approximately 2.5%.^41^ From a hormonal perspective, biopsy of a pheochromocytoma may precipitate a hypertensive crisis, and therefore catecholamine secretion should be excluded prior to tissue sampling. From an oncologic perspective, sampling of a malignant lesion risks seeding the biopsy tract and upstaging the cancer.

With respect to diagnostic performance, a 10-year, retrospective review of all image-guided percutaneous adrenal biopsies performed at a large tertiary care medical center found that within the subset of patients with adrenal incidentalomas with no known extra-adrenal malignancy, the sensitivity of needle biopsy for detecting adrenocortical cancer was only 50%.This contrasts with the groups with known extra-adrenal malignancy or with imaging studies suggesting a non-adrenal primary malignancy, where the probability of an adrenal biopsy specimen being positive for malignancy was 70.6% and 69.0%, respectively.^42^ A more recent systematic review and meta-analysis supported the conclusion that adrenal biopsy is most useful in the diagnosis of adrenal metastasis in patients with extra-adrenal malignancy, with a limited role in patients with adrenal incidentalomas.^41^ Finally, both studies confirm the known difficulties in differentiating between adrenocortical adenoma and carcinoma from a needle biopsy specimen.

Given the poor diagnostic performance of biopsy and the inherent risks, current guidelines suggest that percutaneous adrenal tissue sampling should only be considered in patients with radiographically indeterminate tumors in whom the biopsy results would change management.^18^

## Management and Surveillance

With the increasing incidence of adrenal incidentaloma diagnosis, the cohort of patients with known adrenal nodules continues to expand. For the subset of patients with hormone secretion or suspicion for malignancy, management is typically surgical resection. For patients with non-functional adenomas or indeterminate diagnoses, appropriate surveillance and timing of repeat biochemical testing remain controversial.

Multiple studies have found that while the rate of malignant transformation of a non-functional tumor is rare, previously non-functional tumors can become functional^14,43,44^ The Korean Endocrine Society practice guidelines recommended annual hormone studies for 4–5 years to assess for functionality;^32^ however, the ESE guidelines recommend against repeat hormonal testing in patients with a non-functional tumor at initial evaluation in the absence of new clinical signs of hormone excess.^45^ It is recommended that comorbidities potentially attributable to cortisol excess be reassessed annually. While diagnoses of pheochromocytoma, aldosteronoma, and overt Cushing’s are clear indications for surgery, recommendations around MACS are more nuanced. Ample data support the detrimental physiologic effects of hypercortisolism. Some studies suggest a linear association between cortisol secretion and mortality.^46^ A large, international, retrospective cohort study demonstrated significantly increased cardiometabolic comorbidities and all-cause mortality in patients with adrenal incidentalomas with autonomous cortisol secretion.^47^ However, a large meta-analysis of 32 studies found that while there is an increase in metabolic comorbidities and cardiovascular events in patients with MACS compared with non-functional tumors, there was no significant difference in mortality.^44^ Within the restrictions of these data, current guidelines suggest that patients with MACS secondary to a unilateral adenoma and comorbid conditions associated with hypercortisolism undergo adrenalectomy because of anticipated significant improvements in cardiometabolic health.^18^

## Future Directions

While adrenal incidentalomas are both common and increasing in incidence, there remains a significant gap in understanding and management. Notably, rates of completion of appropriate testing and imaging are low. A systematic review examining adherence to guidelines found that median rates of imaging (34%) and biochemical evaluation (18%) were unacceptably low.^48^ In this study, the factor most consistently associated with improved follow-up was the radiologist’s recommendation, highlighting the significance of radiology reporting and suggesting a potential opportunity for improvement.^48^

Several recent studies have tried to improve identification and management of adrenal incidentalomas by leveraging quality improvement initiatives and the electronic health record (EHR). Studies have targeted different steps to improve follow-up from standardizing radiology reports to engaging primary care providers to EHR-based strategies.^49,50^ Use of a standardized radiology template to report adrenal incidentalomas successfully improved rates of primary care provider (PCP) follow-up, biochemical testing, follow-up imaging, and appropriate specialist referrals in one center.^50^ Direct engagement with PCPs as well as the implementation of multidisciplinary adrenal clinics including endocrinologists and surgeons also represent promising targets for improving follow-up.^51^ One novel approach utilizing a natural language processing algorithm to detect clinically significant adrenal nodules increased biochemical evaluation sevenfold and adrenal-specific imaging threefold in participating patients.^52^ Orthogonal studies in primary aldosteronism utilizing EHR-based tools to automatically identify patients, and active choice nudges to guide provider ordering, resulted in a significantly higher proportion of patients being appropriately screened.^53^

**Fig. 1:**
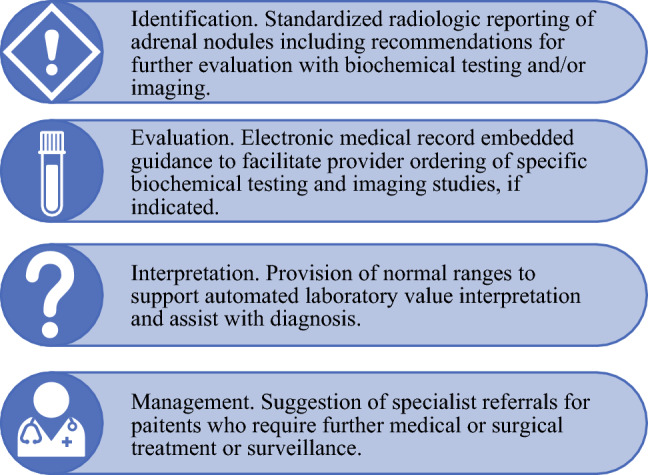
Potential EHR-based interventions to support evaluation and treatment of adrenal incidentalomas. Key steps include identification, evaluation, interpretation, and management, which may be facilitated by EHR-based strategies. *EHR* electronic health record

These novel approaches suggest that there are opportunities to leverage systems-based health care informatics to improve guideline-concordant care for adrenal incidentalomas at multiple levels. Optimal strategies would target: (1) *identification*, using standardized radiologic reporting of adrenal nodules and recommendations for biochemical and imaging evaluation; (2) *evaluation*, using EHR-embedded guidance to facilitate provider ordering of specific biochemical testing and imaging studies if indicated; (3) *interpretation*, providing normal ranges to support laboratory value interpretation and diagnosis; and (4) *management*, facilitating specialist referrals for patients who require further medical or surgical treatment or surveillance, as shown in Fig. 1.

By adhering to established guidelines and leveraging novel informatics-based tools, healthcare providers have an opportunity to ensure effective monitoring and management of adrenal incidentalomas, ultimately improving patient outcomes. Accurate identification of clinically relevant lesions while avoiding unnecessary overtesting will be essential as the detection of adrenal incidentalomas continues to increase in an aging population.
